# The Contingent Prenatal Screening Test for Down’s Syndrome and Neural Tube Defects in West of Iran

**Published:** 2019

**Authors:** Faranak Aghaz, Seyyedeh Zeinab Ojagh, Saber Khanjari, Asad Vaisi-Raygani, Mozafar Khazaei, Mitra Bakhtiari

**Affiliations:** 1- Fertility and Infertility Research Center, Health Technology Institute, Kermanshah University of Medical Sciences, Kermanshah, Iran; 2- Imamreza Clinic, Kermanshah, Iran

**Keywords:** Contingent prenatal screening test, Down syndrome, Iran, Neural tube defects

## Abstract

**Background:**

The purpose of the study was to evaluate the use of contingent prenatal screening for the detection of Down’s syndrome and neural tube defects (NTDs) in west of Iran.

**Methods:**

A prospective study was conducted on 653 pregnant women referred to a medical diagnostic laboratory (Imam Reza Clinic, Kermanshah, Iran) for contingent prenatal screening tests between October 2016 to September 2017.

**Results:**

Among 651 women screened in the first trimester, 8 (1.22%) pregnancies were screen-positive for Down’s syndrome. In the second trimester, among 605 women, 25 (4.13%) had a positive result and all of these women voluntarily underwent amniocentesis. Overall, five pregnancies were complicated with chromosomal abnormalities, including five cases of Down’s syndrome.

**Conclusion:**

In a nutshell, the contingent prenatal screening tests were found to be useful for estimation of Down’s syndrome as well as NTDs in both young and older mothers in west of Iran. These tests should be performed for pregnant women before an invasive test for Down’s syndrome.

## Introduction

According to a report by the World Health Organization (WHO) in 2015, globally, congenital anomalies among Down’s syndrome and neural tube defects (NTDs) are major causes of stillbirths, neonatal death, long-term illness, disability and childhood health problems (1). Despite powerful strides forward in the prenatal diagnosis of congenital anomalies in western countries, mortality rates from hospital-based data in low- and middle-income countries, particularly Iran, for common anomalies have risen to 20–85% (2, 3). It is also well demonstrated that the risk of theses congenital anomalies increases significantly with the mother’s age; the risk is even higher after the age of 35 (4). However, the use of invasive diagnostic tests mainly chorionic villus sampling (CVS) or amniocentesis was introduced in 1970 and of fered to pregnant women of advanced maternal age (5). But the tests require inserting needles in the mother’s abdomen and are known to increase the risk of miscarriage (6). Thus, in order to minimize the risk of miscarriage, contingent prenatal screening tests at the first and/or second trimester of pregnancy are necessary for the prevention of common genetic disorders before invasive testing (7).

Contingent prenatal screening is routinely offered to many pregnant women in the first and second trimester to look for birth defects. In the first trimester, between 11weeks and 6 days (11 w + 6 d) and 13 w+6 d of pregnancy, a combined screening test is performed, which measures the thickness of nuchal translucency (NT) and maternal serum markers such as pregnancy-associated plasma protein A (PAPP-A) and free- beta human chorionic gonadotropin (Free β-hCG) (8, 9). In Down’s syndrome pregnancies, the free β-hCG level is higher and is around 2.0 multiple of the median (MoM), while PAPP-A level is lower and is around 0.5 MoM (10).

In the second trimester, the maternal serum screening is performed around 15–22 weeks of gestation. These blood tests investigate abnormal levels of proteins and hormones such as alpha-fetoprotein (AFP), unconjugated estriol (E3), human chorionic gonadotropin (hCG) and inhibin-A (13). In most western countries, the second trimester test had a higher specificity rate of 80% with a false positive rate of 5% for Down’s syndrome, and also provides useful information for identifying other fetal anomalies such as NTDs (14). Accordingly, in European countries, second trimester screening is currently the most common screening test performed between 15 and 22 weeks of gestation (10, 15).

Despite the accessibility of contingent prenatal screening tests for Down’s syndrome and NTDs, unfortunately no information is available on identifying such tests in Iran, particularly in Kermanshah province. On the other hand, to the best of our insight, there was no distributed information on the effectiveness of prenatal screening biomarkers for congenital anomalies in Iran. Thus, the purpose of the study was to qualify the identifying value of first and second trimester maternal serum screening for the detection of NTDs and Down’s syndrome in the western part of Iran, Kermanshah province.

## Methods

### Subjects:

From October 2016 to September 2017, the contingent prenatal screening test or (first/or second-trimester) was routinely provided for 653 pregnant women who came to Imam Reza clinic. At first, maternal characteristics and medical history were collected from all participants and recorded using a specifically designed form. Then, a computing database was created and registering all information was performed for each enrolled patient and kept confidential by the research team. Furthermore, all of the information including the city of residence, maternal age at time of expected delivery that was divided into three groups (<35, 35–39, and ≥40 years), maternal weight, status of smoking, status of IVF (if done), history of chromosome abnormality in the first degree family, status of disease such as diabetes, thyroids and blood pressure, number of previous gestation, baby sex, being singleton, history of previous abortion, abortion type, proteinuria, position of fetus in uterus, gestational age that was estimated by the measurement of the fetal crown-rump length (16), and serum analysis were registered for each participant. The inclusion criteria included singleton pregnancy of 11 w+6 d and permanent residents of the Kermanshah area. The exclusion criteria were multiple pregnancy, smoking, having IVF baby, other discordant aneuploidies such as trisomy 18 and 13 and in utero fetal death.

### Prenatal screening biomarker assays:

First-trimester risk assessment was provided for Down’s syndrome. The first trimester tests involve measuring fetal nuchal translucency (NT) thickness and all blood samples were collected from patients at 11 w + 6 d and 13 w + 6 d of gestation. Fetal NT measurements were performed at midesagittal plane according to the established criteria published by the fetal medicine foundation of the United Kingdom (11), and compared to NT nomograms at a given gestational age. Increased NT thickness was considered ≥2.5 *mm*. Maternal serum levels of free β-human chorionic gonadotropin (free β-hCG) and plasma protein-A (PAPP-A) were determined simultaneously among all women using ECL (Electro chemiluminescence assay by Cubase E411, Roche Germany), according to the manufacturer’s protocol (Gene med Biotechnologies, South San Francisco, CA, USA). The values of NT, PAPP-A, and free β-hCG were divided by their respective day-specific median levels to determine the multiples of the median of each marker. The women were screened into three groups; one group (high risk screen-positive) was immediately offered a diagnostic test, a second group (screen-negative) received no further screening and a third group was borderline case or lower risk screen positive. Then, the negative and borderline case received second-trimester maternal serum (based on doctor’s diagnosis).

Second-trimester risk assessment was provided for Down’s syndrome and NTDs. Approximately, 605 (92.17%) women also had second-trimester serum analysis to form a contingent test. Risk was then reassessed and those with high risk were offered diagnosis. The second trimester tests involved measuring levels of alpha-fetoprotein (AFP), total chorionic gonadotropin (β-hCG), unconjugated estriol (uE3), and inhibin-A, in maternal serum that were collected from patients at 15 and 22 weeks of gestation. AFP and β-hCG levels were measured using ECL (Electro chemiluminescenceassay by Cubase E411, Roche Germany), uE3 (ELISA method using LDN kit Germany, Dynex two plate, USA) and inhibin A (ELISA method using Beckman-Coulter kit USA, Dynex two plate, USA).

### Confirmation of Down’s syndrome and NTDs:

Oneway ANOVA analysis was applied to each biomarker to obtain reliable multiple of the median. Risk of Down’s syndrome was calculated using Benetech-PRA 3.3.00 software (Logical Medical System, Canada), with a cutoff set at 1:100 for Down’s syndrome and 1:1500 for borderline case. The cases with risk ≥2.5 AFP- MoM for NTDs, advanced age ≥35 years, an extremely high free β-hCG value (MoM ≥10), or abnormal ultrasound findings were referred (based on doctor’s diagnosis) to genetic centers for performing amniocentesis or chorionic villus sampling (CVS) diagnostic tests. Just 5 cases of all Down’s syndrome cases were confirmed by amniocentesis, and all screened subjects received telephone follow-ups until delivery; however, some of the subjects could not be contacted. The number of residual cases (low and borderline risk cases resulted in Down’s syndrome births) was compared, in order to demonstrate the significance of recommending borderline cases-risk cases for further examination.

### Statistical analysis:

The study data were analyzed using SPSS version 23.0 (SPSS variant 23.0, Chicago, IL, USA). This study was approved by the Ethics Committee of Kermanshah University of Medical Sciences. All participants received information on the screening program and provided written consent. Descriptive statistics was used for the analysis of collected data. Continuous variables with normal distribution were described as mean±SD and compared by an independent t test.

## Results

**Figure 1.**
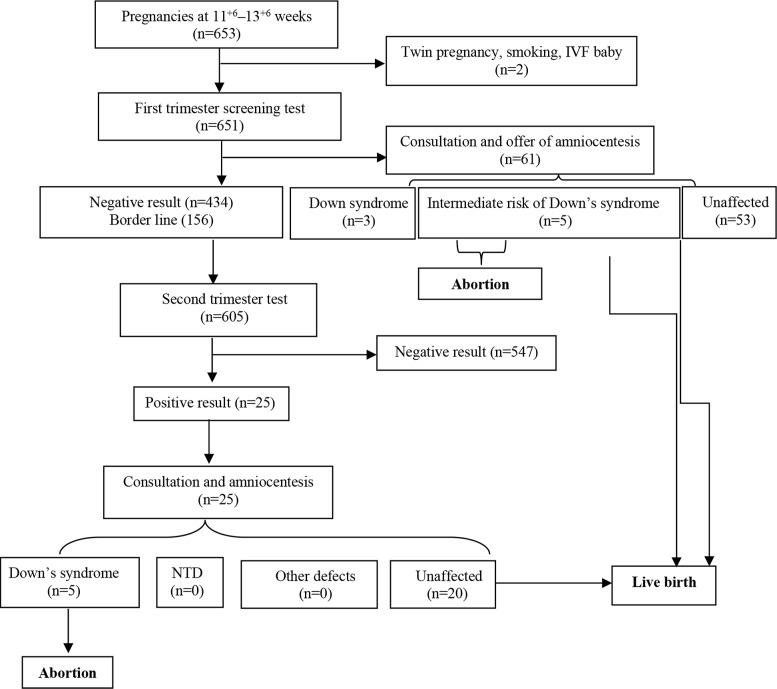
Flowchart for process of screening

During the study period, 653 women at 11 w + 6 d to 13 w + 6 d of pregnancy were referred to Prenatal Diagnosis Center, Imam Reza Clinic, Kermanshah, Iran, for prenatal care. Two women were subsequently excluded due to smoking and IVF baby; the remaining 651 women met the eligible criteria and agreed to undergo the contingent prenatal screening test. Among 653 women, 168 (25.72%) had a borderline case, 49 (7.5%) had positive first trimester test for Down’s syndrome and 434 (66.66%) had unaffected pregnancies (negative result) during first trimester pregnancy. During the second trimester screening test, 605 (92.64%) women agreed to undergo the quadruple test. Among them, there were 58 cases (9.25%) with second screening positive result (33 and 25 cases were positive for Down’s syndrome and NTDs, respectively) and 549 cases (90.74%) with second screening had negative result. Finally, among 58 cases, 28 cases refused amniocentesis and continued pregnancy, and 5 cases with chromosomal abnormality were identified as shown in figure 1.

Table 1 shows demographics of the 653 women screened. The mean maternal age and maternal weigh were 30.15±5.20 years and 68.07±11.26 kg, respectively. In this study, maternal age was significantly different so congenital anomalies were evaluated in three age groups. Therefore, the mean gestational age was 273.59±12.26, 268.27± 9.77, and 268.88±14.20 days, respectively in three age groups (Table 1).

**Table 1.** Demographics of the 653 women screened

**Maternal demographics**	**(Mean±SD)**	**p-value**
**Median maternal age (years)**	30.15±5.20^*^	---
**Median gestational age (days)**		
≤35 years (n=498)	273.59±12.26	0.024
35–39 years (n=107)	268.27±9.77	0.485
≥40 years (n=46)	268.88±14.20	0.608
**Median weight (*kg*)**	68.07±11.26	---

Data are presented as absolute values (mean±SD)

The characteristics of the pregnancies affected and unaffected by Down’s syndrome are given in table 2. Overall, the first trimester test yielded 217 (33.33%) high risk cases in which 168 (77.41%) were borderline cases for Down’s syndrome and 49 (22.58%) had Down’s syndrome and very low risk (negative result) cases of the screening were 434 (66.66%). Among the 168 borderline cases for Down’s syndrome, 102 were≤35 years (60.71% [124/168]), 44 aged 35–39 years (26.19% [44/168]), and 22 were ≥40 years or older (13.09% [22/168]). Among the 49 women with the Down’s syndrome, 15 were ≤35 years (30.61% [15/49] of age group), 18 aged 35–39 years (34.78% [18/49]), and 16 were ≥40 years (36.73% [16/49]). The MoM levels of PAPP and Free β-hCG were significantly different in the borderline cases of Down’s syndrome (≤35 years) and cases with Down’s syndrome (in three age groups) compared with the unaffected pregnancy (Table 2). The NTMoM marker showed older ages in Down’s syndrome pregnancy (in three age groups) compared with the unaffected pregnancy (Table 2).

**Table 2.** Characteristics of pregnancyaffected by Down’s syndrome and borderline case and unaffected pregnancies in first trimester screening test

**Characteristics**	**Maternal age, years**	**Unaffected pregnancy**	**Borderline case**	**Down’s syndrome pregnancy**	**p-value**
**PAPP-MoM**					
	<35 (n=498)	382 (1.29±0. 87)	102 (0.88±0.85)^*^	15 (0.64±0.52)^*^	0.000
	35–39 (n=107)	43(1.55±0.90)	44 (1.15±0.86)	18 (0.72±0.49)^*^	0.002
	≥40 (n=46)	9(1.58±0.54)	22 (1.18±0.68)	16 (0.55±0.32)^*^	0.000
**free β-hCG-MoM**					
	<35 (n=498)	382 (1.22±0. 90)	102 (2.23±1. 18)^*^	15 (3.92±1. 73)^*^	0.037
	35–39 (n=107)	43 (1.14±0.93)	44 (1.17±1.07)	18 (6.11±1.31)^*^	0.007
	≥40 (n=46)	9 (0.82±0.37)	22 (1.34±0.88)^*^	16 (1.98±1.23)^*^	0.017
**NT-MoM**					
	<35 (n=498)	382 (1.15±0. 24)	102 (1.26±0. 38)^*^	15 (1.86±0.88)^*^	0.000
	35–39 (n=107)	43 (1.12±0.20)	44 (1.16±0.24)	18 (1.41±0.25)^*^	0.000
	≥40 (n=46)	9 (1.04±0.26)	22 (1.18±0.22)^*^	16 (1.27±0.18)^*^	0.050

Data are presented as absolute values n (Mean±SD) and p-value. The superscript

* indicate significant differences among experimental groups (p≤0.05). MoM; multiple of the median, PAPP; pregnancy-associated plasma protein A, free β-hCG; free-beta human chorionic gonadotropin

Table 3 provides the characteristics of the unaffected, Down’s syndrome and Neural tube defects (NTDs) pregnancies in second trimester screening test. Among the 549 women with a negative test, 453 were ≤35 years (82.51% [453/549] of age group), 71 aged 35–39 years (12.93% [71/549]) and 25 were ≥40 years (4.55% [25/549]). Among the 58 women with a positive second trimester test, 31 and 25 cases were positive for Down’s syndrome and NTDs, respectively. Sixteen cases were ≤35 years for both NTDs (64% [16/25]) and Down’s syndrome (51.61% [16/31]), 11 and 7 cases aged 35–39 years for Down’s syndrome (35.48% [11/31]) and NTDs (28% [7/25]), respectively and 4 and 2 cases were ≥40 years for NTDs and Down’s syndrome (12.90% [4/31] and 8 % [2/25], respectively) (Table 3). There was significant difference in the MoM value for AFP between Down’s syndrome (aged 35–39 years) and NTDs (three age groups) compared with unaffected pregnancies. The uE3 MoM values in 35–39 and ≥40 years groups for Down’s syndrome and ≤35 years group for NTDs pregnancies were significantly lower than the unaffected pregnancy. The MoM values for β-hCG and inhibin A were not significantly higher in Down’s syndrome and NTDs pregnancies than in unaffected pregnancies except ≤35 years group.

**Table 3.** Characteristics of pregnancy affected by Down’s syndrome, neural tube defects and unaffected pregnancy in second trimester screening test

**Characteristics**	**Maternal age, y**	**Unaffected pregnancy**	**Down’s syndrome pregnancy**	**NTDs pregnancy**	**p-value**
**β-hCG-MoM**					
	<35 (n=485)	453 (1.25±0. 64)	16 (2.63±1.25)^*^	16 (2.13±1.42)^*^	0.000
	35–39 (n=89)	71 (1.29±0.76)	11(1.94±0.89)^*^	7 (1.39±1.07)	0.049
	≥40 (n=31)	25 (1.03±0.51)	4 (1.36±0.51)	2 (0.94±0.35)	0.450
**AFP-MoM**					
	<35 (n=485)	453 (1.21±0. 92)	16 (1.04±0. 50)	16 (2.65±1. 32)^*^	0.000
	35–39 (n=89)	71 (1.26±0.48)	11(0.81±0.30)^*^	7 (2.39±1.21)^*^	0.000
	≥40 (n=31)	25(1.19±0.28)	4 (0.80±0.26)	2 (1.97±0.89)^*^	0.001
**uE3-MoM**					
	<35 (n=485)	453 (1.01±0. 36)	16 (0.67±0. 31)	16 (1.02±0. 32)^*^	0.001
	35–39 (n=89)	71 (0.99±0.35)	11(0.66±0.18)^*^	7 (0.83±0.32)	0.010
	≥40 (n=31)	25 (1.04±0.35)	4 (0.50±0.15)^*^	2 (0.89±0.16)	0.020
**Inhibin A- MoM**					
	<35 (n=485)	453 (1.12±0. 50)	16 (1.89±0. 53)^*^	16 (1.61±1. 12)^*^	0.000
	35–39 (n=89)	71 (1.19±0.88)	11 (1.50±0.51)	7 (1.30±0.69)	0.050
	≥40 (n=31)	25 (1.06±0.45)	4 (1.20±0.43)	2 (1.34±0.12)	0.542

Data are presented as absolute values n (Mean±SD) and p-value. The superscript

* indicate significant differences among experimental groups (p≤0.05). NTDs; neural tube defects, MoM; multiple of the median, AFP; α-fetoprotein, β-hCG; beta-human chorionic gonadotropin, uE3; unconjugated estriol

In total, 13 cases of aneuploidies were detected in this study by amniocentesis, that all of them had Down’s syndrome (1 case in 150). The other 73 women who voluntarily underwent amniocentesis had an unaffected pregnancy (first and second trimester). All studied women were followed up until delivery.

## Discussion

According to the March of Dimes (MOD) report, about 94% of total congenital anomalies occur in low-and middle-income countries (3, 17). Also, it has recently been reported that prevalence of congenital anomalies such as open neural tube defects (NTDs) and Down’s syndrome (also known as Trisomy 21or DS) in Iran is slightly higher than that reported in countries from the Middle-East region (18–20). It is well also demonstrated that different factors of age, race and ethnicity can potentially change the rates of congenital anomalies in different regions of countries (21). Therefore, the beneficial effect of the prenatal screening at a national level should be evaluated before worldwide implementation. Hence, for the first time, the effectiveness of the first trimester and second trimester screening biomarkers used as aneuploidy screening was assessed especially for diagnosing Down’s syndrome and NTDs in west of Iran.

In Kermanshah province at prenatal screening centers, the first-trimester screening is based on maternal age, fetal NT and biochemical testing is then performed for determining high and borderline risk cases of Down’s syndrome. In this study, the first trimester screening of 653 pregnant women detected 49 cases of Down’s syndrome and 168 borderline cases of Down’s syndrome. Our result of prenatal screening for Down’s syndrome during the first trimester showed that the MOM values of free beta-human chorionic gonadotropin (free β-hCG) and pregnancy- associated plasma protein A (PAPP-A) (both in unaffected pregnancies and three age groups) were higher than those which had been reported by the western countries (22), but were similar to a systematic review and meta-analysis of a predominantly Chinese case (23). Based on our findings, in Down’s syndrome pregnancies, the mean MoM value decreased to 0.64, 0.72 and 0.55 for PAPP-A, and increased to 3.92, 6.11 and 1.98 for free β-hCG in ≤35, 35–39 and ≥40 age-groups, respectively. In other words, advanced maternal age also increases the risk of some chromosomal abnormalities including Down’s syndrome, which is similar to reports of other countries according to World Health Organization (WHO) reports (24). Also, our result showed that fetal NT- MOM values for borderline cases of Down’s syndrome and Down’s syndrome pregnancies were significantly higher than unaffected pregnancy cases in three age groups, which means that its higher values will significantly increase the final risk of Down’s syndrome. This finding is consistent with other investigations which reported the detection rate in western countries (3, 25).

In Kermanshah province prenatal screening centers, the second trimester screening is based on second trimester and biochemical testing is then performed for determining high and borderline risk cases of Down’s syndrome and NTDs. In the present study, 605 women received the second trimester test in which 31 Down’s syndrome and 24 NTDs cases were detected. Our result of second trimester test for unaffected pregnancies during the second trimester showed that the MoM values of human chorionic gonadotropin (β-hCG), α-fetoprotein (AFP), unconjugated estriol (uE3) and inhibin A in ≤35, 35–39 and ≥40 years age groups (Table 3) were higher than those which had been reported by the western countries (26). Based on our results, Down’s syndrome pregnancy is associated with high levels of β-hCG, uE3 and inhibin A and low levels of AFP and uE3, which is completely in agreement with the results of western countries (26), and in NTDs pregnancies, the AFP MoM values were significantly higher than unaffected pregnancy in three age groups, which means that its higher values will significantly increase the final risk of NTDs, which is similar to a report of other countries (27).

Also, the prevalence of Down’s syndrome in 35–39 and ≥40 years age groups was almost twice the prevalence of Down’s syndrome in fetuses of mothers ≤35 years, although the frequency of Down’s syndrome was higher in mothers ≤35 years of age, which is similar to the reports of western countries (26). The prevalence of Down’s syndrome in this study was one case in 150 (5 in 653) and the rate is slightly higher than the value in the study published in 2015 (prevalence of Down’s syndrome of 1 per 491 in Tehran, Iran) (25). There were no cases of NTDs in the three age groups of the present study population; this finding isn’t consistent with Pasha et al.’s systematic review in 2017 (20). However, the small number of participants in this study might have affected these outcomes. The accuracy of second trimester test was higher than first trimester test in three age group, which confirms the highest effectiveness of second trimester test in clinical settings in Kermanshah province.

In this study, there were only 653 participants, which might be considered a small sample and might not reflect the general women pregnancy population; therefore, no strong recommendation can be offered about the best test and future researchers cannot generalize their results.

## Conclusion

In a nutshell, evidence from the present study revealed that the intermediate risk of Down’s syndrome compared with Down’s syndrome in first trimester and the second trimester needs the use of accurate prenatal diagnostic tests. Therefore, in intermediate cases, first trimester test and also the second trimester test may be recommended for use as a feasible and beneficial tool for Down’s syndrome screening in countries with high rates of congenital anomalies, before an invasive screening test. First trimester and the second trimester tests for intermediate risk of Down’s syndrome compared with Down’s syndrome have the highest identification rate in three age groups which confirms the effectiveness of these two tests in clinical settings in Kermanshah province in west of Iran.
